# Supplementary material for: Epithelial-to-Mesenchymal Transition Is Not a Major Modulating Factor in the Cytotoxic Response to Natural Products in Cancer Cell Lines
Source: Molecules. 2021 Sep 27;26(19):5858. doi: 10.3390/molecules26195858 (PMC8512490; doi:10.3390/molecules26195858)
Supplement: Supplementary file 1 [file molecules-26-05858-s001.zip › molecules-1326234-supplementary.pdf]

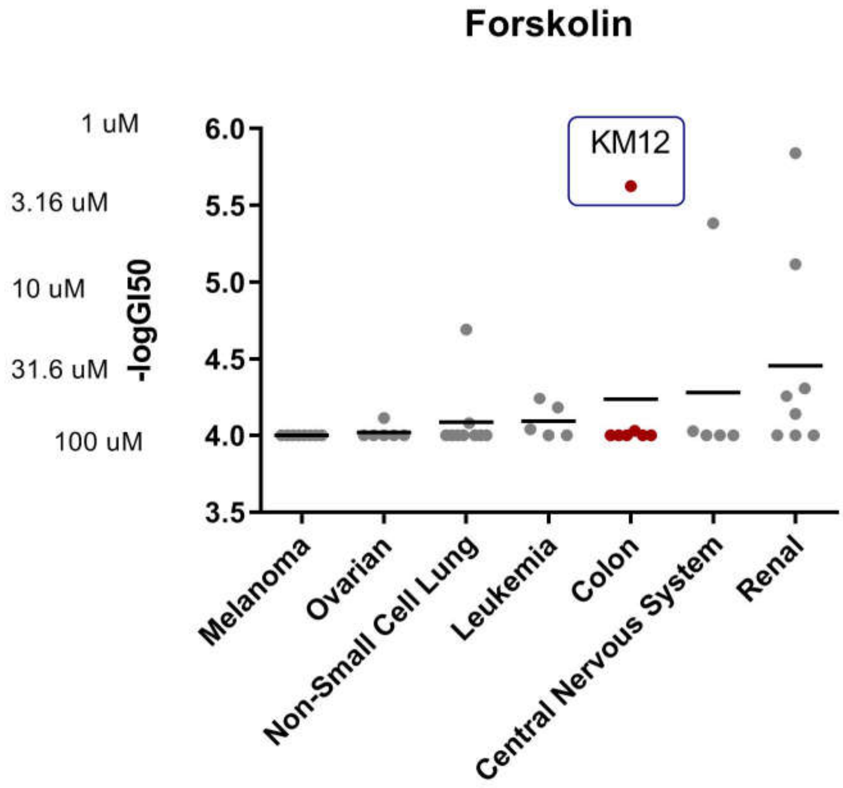

Figure S1. Growth inhibition screening assay data in NCI-60 for forskolin in melanoma, ovarian, lung, leukemia, colon, central nervous system, and renal cancer cell lines

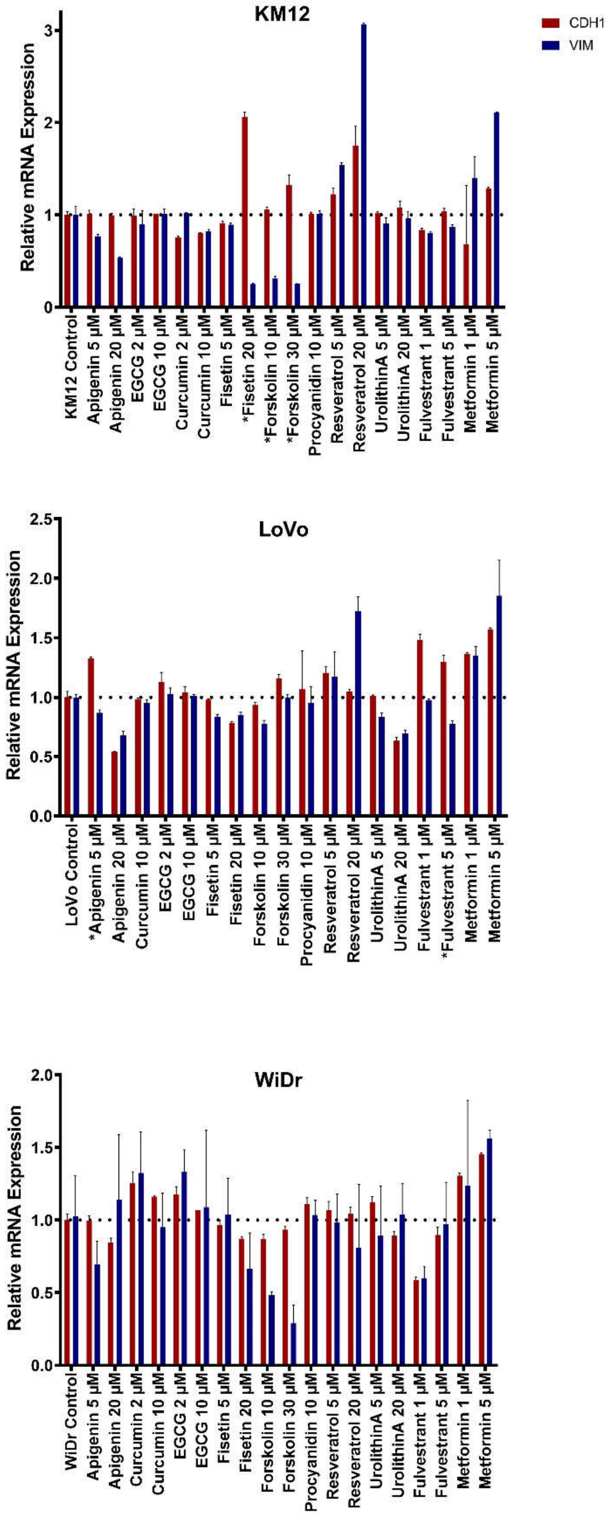

Figure S2. Effect of natural compounds and repurposed agents on the expression of EMT marker genes CDH1 and VIM IN LoVo, KM12, and WiDr colon cancer cell lines.

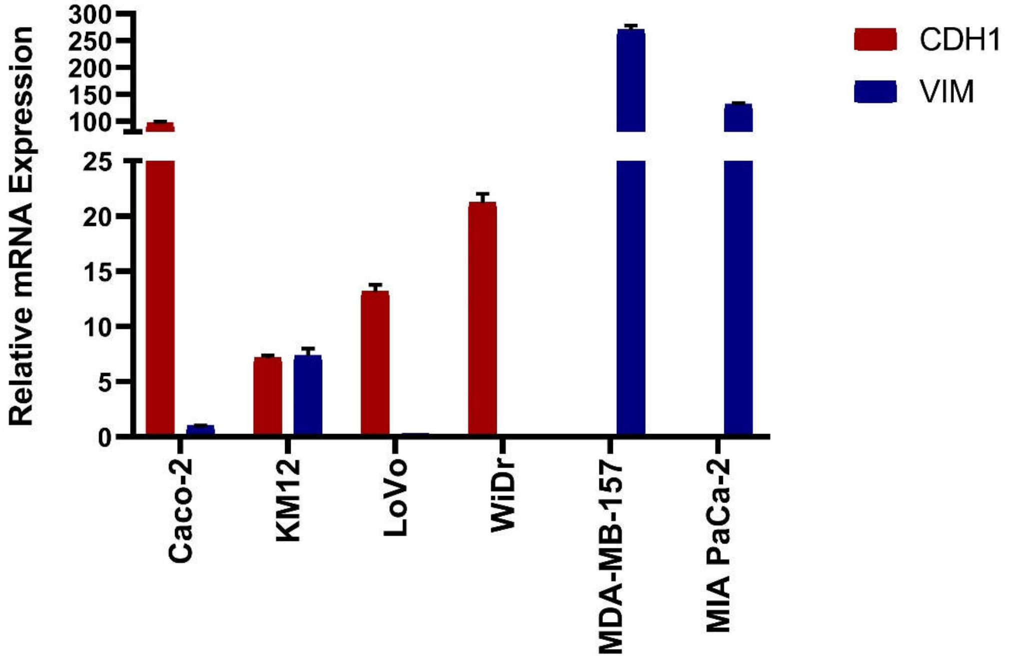

Figure S3. Basal expression levels of CDH1 and VIM genes in Caco-2, KM12, LoVo, WiDr, MDA-MB-157, and MIA PaCa-2 cancer cell lines by qRT-PCR.

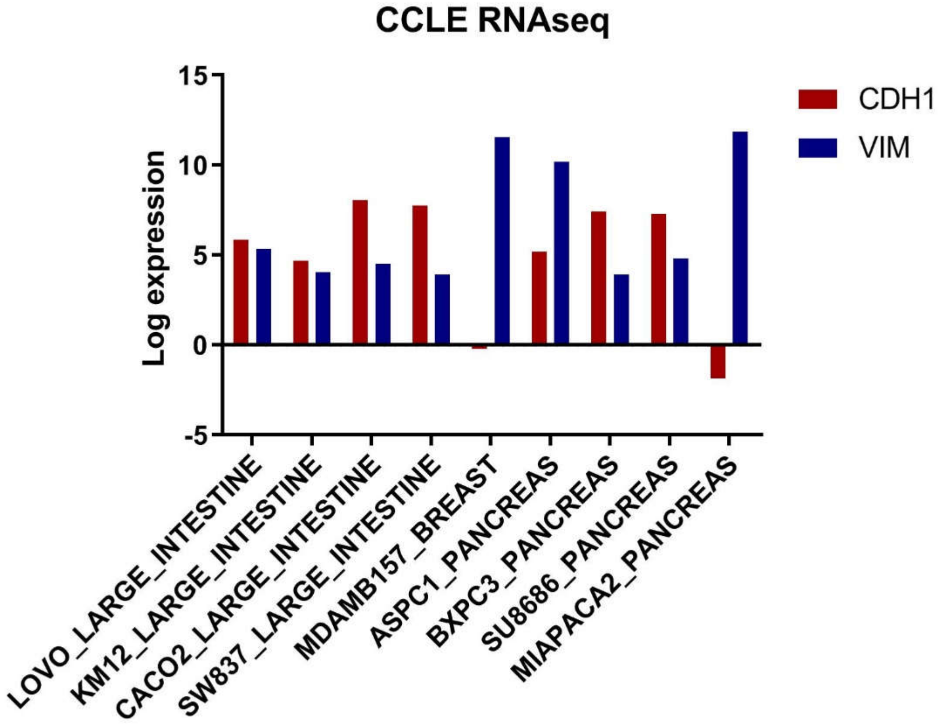

Figure S4. CCLE RNA-seq expression levels of CDH1 and VIM genes in LoVo, KM12, SW837, MDA-MB-157, AsPC-1, BxPC-3, MIA PaCa-2, and SU8686 cell lines

Table S1. Overall analysis of selective MET induction by natural products in colon, breast and pancreatic cancer cell lines.

	Induction of MET					
	LoVo	WiDr	Caco2	KM12	MDA-MB-157	MIA PaCa-2
Apigenin	yes	no	no	no	no	partial
Curcumin	no	no	no	no	no	no
EGCG	no	no	no	no	no	partial
Fisetin	no	no	no	yes	no	partial
Forskolin	no	no	no	yes	no	partial
Procyanidin B2	no	no	no	no	no	partial
Resveratrol	no	no	no	no	yes	partial
Urolithin A	no	no	no	no	no	partial
Fulvestrant	yes	no	no	no	no	partial
Metformin(mM)	no	no	no	no	no	partial

*yes: it indicates upregulation in CDH1 and downregulation in VIM expression.
* partial: it suggests that treatments resulted in mild inductions, only upregulation in CDH1.

Table S2. Effects of natural product treatments alone or in combination with chemotherapeutics on cell viability and analysis of combination index in KM12, WiDr and SW837 colon cancer cell lines;

KM12

Apigenin	0 µM		0.2 µM		2 µM		5 µM		10 µM	
FU 0 µM	99.28206	100.7179	102.4211	103.5077	99.34349	99.16031	99.85319	99.33999	90.71112	86.06503
FU 1 µM	94.97785	94.20457	97.60434	95.11749	94.77475	96.74047	96.46273	102.2315	87.70083	80.33601
FU 10 µM	57.87597	58.45969	59.06661	59.22307	60.52919	58.12396	65.88253	62.2792	61.54413	61.61637

Dose FU	Dose Apigenin	Effect	CI
1	2	0.04	1.53
1	5	0.01	5.94
1	10	0.16	1.17
10	2	0.41	1.15
10	5	0.36	1.54
10	10	0.39	1.69

Curcumin	0 µM		0.1 µM		0.5 µM		2 µM		5 µM	
FU 0 µM	98.74288	101.2571	102.2016	102.573	102.6252	105.506	99.81693	99.78471	97.21125	98.27392
FU 1 µM	94.37184	93.05855	99.72137	99.76099	98.6495	99.3974	98.44136	94.59409	92.45408	91.51159
FU 10 µM	53.51187	55.21242	56.50993	56.38076	57.45232	59.94544	52.35322	58.91612	63.67348	60.96987

Dose FU	Dose Curcumin	Effect	CI
1	2	0.04	2.23
1	5	0.08	1.54
10	2	0.44	1.14
10	5	0.38	1.61

EGCG	0 µM		0.2 µM		1 µM		6 µM		12 µM	
FU 0 µM	100.2122	99.78781	100.3999	103.8237	100.4802	106.1643	99.48477	98.84684	94.35282	89.77618
FU 1 µM	102.0889	105.4039	109.8305	108.9807	110.692	108.4059	103.7998	105.4015	93.59078	88.79344
FU 10 µM	63.72736	66.45627	66.2711	69.01405	66.67085	65.46143	65.08788	65.41448	69.94951	65.52053

Dose FU	Dose EGCG	Effect	CI
1	6	0.00	52.96
1	12	0.09	1.56
10	6	0.35	1.31
10	12	0.32	1.75

Fisetin	0 µM		0.2 µM		2 µM		10 µM		20 µM	
FU 0 µM	99.52548	100.4745	102.5777	100.014	101.1294	98.48324	78.81386	77.06842	50.51539	48.61358
FU 1 µM	86.82409	88.81246	91.7443	91.43819	93.05246	90.98627	70.31589	71.00478	49.94571	47.89199
FU 10 µM	55.80934	58.34945	57.32975	56.30971	56.97477	57.00658	57.48886	56.35719	48.9897	50.42797

Dose FU	Dose Fisetin	Effect	CI
1	2	0.08	2.16
1	10	0.29	0.98
1	20	0.56	1.10
10	2	0.43	1.12
10	10	0.43	1.61
10	20	0.55	1.57

Forskolin	0 µM		0.1 µM		1 µM		4 µM		10 µM	
FU 0 µM	97.08199	102.918	94.87923	95.9937	77.47949	77.22055	51.46381	47.34839	26.10205	20.02818
FU 1 µM	85.85008	87.51428	86.74709	88.33636	70.24636	64.24607	40.90244	34.91566	27.53057	25.53775
FU 10 µM	51.17722	56.47757	50.55625	52.18259	26.29085	32.02568	20.41225	18.1795	12.63835	12.80792

Dose FU	Dose Forskolin	Effect	CI
1	0.1	0.13	1.34
1	1	0.33	0.91
1	4	0.62	0.75
1	10	0.74	0.99
10	0.1	0.49	0.91
10	1	0.71	0.37
10	4	0.81	0.37
10	10	0.87	0.41

Procyanidin B2	0 µM		0.2 µM		2 µM		5 µM		10 µM	
FU 0 µM	98.21213	101.7879	105.1714	102.5099	102.5638	105.9557	113.1331	111.1022	111.3244	111.134
FU 1 µM	93.43693	101.4472	101.0375	101.7402	99.73338	100.8318	101.5975	101.4047	98.86807	102.9801
FU 10 µM	63.02518	65.28795	64.00622	64.29634	63.96634	61.65706	63.28017	62.51873	61.64567	60.66789

not applicable

Resveratrol	0 µM		0.2 µM		1 µM		5 µM		10 µM	
FU 0 µM	99.29318	100.7068	100.153	98.96305	99.55018	102.0202	100.3202	98.0349	86.03089	98.6158
FU 1 µM	91.63867	95.92044	99.15304	98.06305	99.02018	96.25069	93.03678	93.02929	86.03089	85.70164
FU 10 µM	59.26963	58.76849	63.85584	60.89824	63.95409	63.01816	64.63085	68.3666	64.63038	63.49528

Dose FU	Dose Resveratrol	Effect	CI
1	5	0.07	1.40
1	10	0.14	1.21
10	5	0.34	1.65
10	10	0.36	1.77

Urolithin A	0 µM		0.2 µM		2 µM		10 µM		20 µM	
FU 0 µM	100.4503	99.54969	97.94841	95.63498	92.73793	96.37025	99.87809	97.44059	98.30483	98.48381
FU 1 µM	91.34569	92.08693	91.26599	91.4913	90.2753	92.89743	87.47294	90.47565	88.91344	88.97326
FU 10 µM	55.35002	54.58313	51.6162	49.6386	49.35035	49.06181	51.83511	53.58291	55.04214	52.81352

Dose FU	Dose Urolithin A	Effect	CI
1	0.2	0.09	1.21
1	2	0.09	1.18
1	10	0.11	0.89
1	20	0.11	0.90
10	0.2	0.49	0.82
10	2	0.51	0.77
10	10	0.47	0.90
10	20	0.46	0.95

Fulvestrant	0 µM		0.2 µM		1 µM		2 µM		5 µM	
FU 0 µM	99.60805	100.392	89.11045	98.97392	107.9001	103.3964	102.4515	99.52464	93.13578	91.31921
FU 1 µM	96.88598	100.5147	99.41956	99.38907	99.98798	94.15472	89.7069	90.02975	75.76506	73.48055
FU 10 µM	55.57249	55.7913	57.60556	55.9437	54.00655	55.39776	52.51997	53.47536	45.64492	44.82476

Dose FU	Dose Fulvestrant	Effect	CI
1	0.2	0.01	1.96
1	5	0.25	0.24
10	0.2	0.43	1.02
10	5	0.55	0.80

Metformin	0 µM		0.1 mM		0.5 mM		1 mM		4 mM	
FU 0 µM	100.2145	99.78545	100.0377	109.5112	102.7132	103.2114	88.36901	92.47161	71.9229	71.48095
FU 1 µM	99.03275	99.25646	109.3814	105.4517	103.3302	104.2203	91.97882	85.13193	75.104	70.30712
FU 10 µM	61.86235	63.91288	57.69234	58.64721	56.55932	55.49098	51.15081	48.76971	51.03952	43.75988

Dose FU	Dose Metformin	Effect	CI
1	1	0.11	0.88
1	4	0.27	1.05
10	1	0.50	0.83
10	4	0.53	1.02

WiDr

Apigenin

	0 µM		0.2 µM		2 µM		5 µM		10 µM	
FU 0 µM	98.05215	101.9479	98.12489	99.07334	89.2554	96.54399	92.00587	90.54227	90.67463	90.11359
FU 1 µM	87.56095	88.15706	84.85107	86.22521	89.64099	85.20773	86.62741	83.79545	77.90979	77.40193
FU 10 µM	43.45142	45.81624	45.29724	45.2452	47.02319	46.80631	45.81282	48.24446	47.81992	46.27354

Dose FU	Dose Apigenin	Effect	CI
1	5	0.15	0.78
1	10	0.22	0.46
10	5	0.53	1.09
10	10	0.53	1.09

Curcumin

	0 µM		0.1 µM		0.5 µM		2 µM		5 µM	
FU 0 µM	94.31725	105.6827	105.0954	97.16378	102.2803	104.1871	103.0836	103.8179	95.62278	96.60406
FU 1 µM	89.90298	88.58253	92.18415	90.15578	92.83583	93.24259	90.026	90.02355	83.04935	82.70242
FU 10 µM	46.47987	47.53016	51.24495	49.6834	54.37767	49.88028	54.17662	57.1543	51.89862	49.30851

not applicable

EGCG

	0 µM		0.2 µM		1 µM		6 µM		12 µM	
FU 0 µM	100.4066	99.5934	94.78986	100.7441	98.01673	98.82008	90.03274	79.78179	91.81461	83.39743
FU 1 µM	84.21745	83.36015	91.38931	88.33447	82.48617	82.55425	78.50819	74.44973	58.88165	58.67684
FU 10 µM	53.0206	61.04164	62.13877	57.25965	65.73931	62.59399	59.09795	60.55218	48.59021	44.36569

Dose FU	Dose EGCG	Effect	CI
1	1	0.18	0.94
1	6	0.24	0.88
1	12	0.41	0.52
10	1	0.36	1.70
10	6	0.40	1.44
10	12	0.54	0.75

Fisetin

	0 µM		0.2 µM		2 µM		10 µM		15 µM	
FU 0 µM	100.369	99.63104	95.96081	92.84244	91.53608	93.7319	92.58253	91.05559	93.34981	89.87765
FU 1 µM	79.21507	78.50319	76.25834	77.90293	78.28739	76.74931	76.59097	77.6568	75.90768	76.05632
FU 10 µM	45.53616	40.68051	43.63015	44.35359	46.60986	44.82563	44.58753	44.92258	43.74157	42.62548

Dose FU	Dose Fisetin	Effect	CI
1	10	0.23	0.85
1	15	0.24	0.78
10	10	0.55	1.12
10	15	0.57	1.00

Forskolin

	0 µM		0.1 µM		1 µM		4 µM		10 µM	
FU 0 µM	100.101	99.89895	94.60385	93.57781	83.56756	92.99683	92.4641	93.02005	92.00066	86.03562
FU 1 µM	64.38815	63.53472	60.17345	63.65186	64.60879	60.81437	79.20026	80.58451	79.18328	75.80129
FU 10 µM	42.94758	41.03679	43.07401	36.92637	44.03767	43.4756	44.01534	40.66154	43.49138	41.43497

Dose FU	Dose Forskolin	Effect	CI
1	5	0.20	10.16
1	10	0.23	6.97
10	5	0.58	1.02
10	10	0.58	1.09

Procyanidin B2

	0 µM		0.2 µM		2 µM		5 µM		10 µM	
FU 0 µM	101.2972	98.70278	108.6027	101.2461	102.3746	102.2047	98.75472	100.5856	105.3271	99.18554
FU 1 µM	84.21635	87.89606	88.56997	88.40746	89.72067	90.42351	87.64379	89.45167	87.1262	85.41221
FU 10 µM	52.16328	52.15183	54.76686	54.44729	54.26812	54.83302	54.24585	52.90729	53.92603	48.55088

not applicable

Resveratrol

	0 µM		0.2 µM		1 µM		5 µM		10 µM	
FU 0 µM	98.09586	101.9041	101.1795	103.3645	105.3169	103.0131	101.5102	96.61453	95.24443	92.8172
FU 1 µM	82.38939	83.57975	85.7393	96.6787	97.86687	98.49778	97.18144	94.63898	92.93741	95.36033
FU 10 µM	47.08167	47.02963	45.22623	46.74354	47.83219	45.16235	46.54853	45.96148	43.06797	42.06882

Dose FU	Dose Resveratrol	Effect	CI
1	5	0.04	9.17
1	10	0.06	5.83
10	5	0.54	1.13
10	10	0.57	1.12

Urolithin A

	0 µM		0.2 µM		2 µM		10 µM		20 µM	
FU 0 µM	99.63377	100.3662	102.0161	101.8795	99.88065	101.7037	98.71302	100.8445	95.2057	94.144
FU 1 µM	77.73499	79.77949	81.90459	82.96385	86.11031	87.8259	83.18611	81.62813	80.35842	76.0906
FU 10 µM	33.86413	36.09638	37.49825	37.99773	35.32719	38.77957	37.81187	37.72863	36.55698	35.73084

Dose FU	Dose Urolithin A	Effect	CI
1	10	0.18	1.67
1	20	0.22	1.63
10	10	0.62	1.41
10	20	0.64	1.53

Fulvestrant

	0 µM		0.2 µM		1 µM		2 µM		5 µM	
FU 0 µM	98.33455	101.6654	99.5742	99.93843	100.0203	99.03372	94.91252	91.53761	87.10711	81.1884
FU 1 µM	90.9436	89.75387	87.77736	93.08727	90.8119	92.6968	88.46621	81.55128	73.7717	73.04137
FU 10 µM	41.98735	40.07409	44.66217	42.39088	48.47714	39.07385	36.34389	38.56358	32.70248	32.21891

Dose FU	Dose Fulvestrant	Effect	CI
1	2	0.15	0.95
1	5	0.27	0.58
10	2	0.63	0.88
10	5	0.68	0.74

Metformin

	0 mM		0.1 mM		0.5 mM		1 mM		4 mM	
FU 0 µM	99.09721	100.9028	105.2088	111.4602	107.5675	101.6401	96.17197	102.3612	89.53257	84.34765
FU 1 µM	77.5122	82.68096	85.8325	81.04934	77.45965	76.00471	75.46011	74.92547	75.25527	73.377
FU 10 µM	36.68754	38.99299	38.80336	37.19238	36.42897	36.66671	34.84144	34.65884	32.42591	29.71203

Dose FU	Dose Metformin	Effect	CI
1	1	0.25	0.87
1	4	0.26	1.32
10	1	0.65	0.91
10	4	0.69	0.94

SW837

	0 µM		1 µM				10 µM				Apigenin	Dose Irinotecan	Dose Apigenin	Effect	CI
Irinotecan 0 µM	97.15299	102.1417	99.59676	101.3458	97.19682	98.92857	77.6426	81.24057	83.64891	89.06951		2	1	0.12	0.74
Irinotecan 2 µM	88.83328	94.39701	92.49201	90.51686	85.76641	84.68103	68.18822	78.62785	78.2027	82.09867		2	10	0.23	0.96
Irinotecan 40 µM	49.78258	53.01504	48.93643	50.88786	51.51288	51.3654	47.59846	50.57213	51.49618	52.16275		40	1	0.49	1.01
												40	10	0.49	1.29

	0 µM		1 µM				10 µM				Curcumin	Dose Irinotecan	Dose Curcumin	Effect	CI
Irinotecan 0 µM	98.80703	101.193	98.73655	97.51373	100.0016	103.4618	61.50752	59.22039	55.71983	55.73871		2	1	0.00	76.65
Irinotecan 2 µM	94.60058	94.87514	100.3086	106.8806	104.6325	100.7834	62.46111	63.26986	60.35884	59.29891		2	10	0.39	1.11
Irinotecan 40 µM	54.84712	55.51192	63.28796	62.94656	57.97031	57.12351	28.14772	33.22373	31.74641	29.31972		40	1	0.40	1.35
												40	10	0.69	1.01

	0 µM		2 µM				20 µM				EGCG	Dose Irinotecan	Dose EGCG	Effect	CI
Irinotecan 0 µM	99.59184	101.1681	92.53342	92.41268	100.7044	93.6989	6.321786	1.826041	2.037467	1.349718		2	2	0.11	1.29
Irinotecan 2 µM	91.73329	94.09121	86.93334	92.81906	89.68102	85.51231	7.419181	8.402556	8.295844	7.271754		2	20	0.92	1.45
Irinotecan 40 µM	54.83873	48.14484	57.7426	52.42539	46.29079	46.21267	2.486829	3.308297	1.987221	2.39446		40	2	0.49	1.33
												40	20	0.97	1.02

	0 µM		1.5 µM				15 µM				Fisetin	Dose Irinotecan	Dose Fisetin	Effect	CI
Irinotecan 0 µM	97.15416	102.8458	97.39657	103.4176	96.28276	102.6129	79.73326	81.56588	83.45795	82.13376		2	1.5	0.04	2.42
Irinotecan 2 µM	91.93948	94.94662	97.08871	95.42689	98.25464	95.28641	73.41732	73.87462	73.88626	73.57898		2	15	0.26	0.97
Irinotecan 40 µM	49.14735	55.95867	58.81978	58.18875	53.60002	55.11166	40.24218	41.25238	39.15208	40.51265		40	1.5	0.44	1.21
												40	15	0.60	0.99

	0 µM		1 µM				10 µM				Forskolin	Dose Irinotecan	Dose Forskolin	Effect	CI
Irinotecan 0 µM	99.93154	100.7738	102.4432	100.9178	98.85865	96.21906	100.7157	99.72174	103.1117	93.53251		2	1	0.05	1.74
Irinotecan 2 µM	92.77652	93.59734	94.0929	96.10494	94.6818	95.15	93.56451	88.40009	99.22251	92.71353		2	10	0.07	1.69
Irinotecan 40 µM	48.24391	52.13215	58.3285	60.35967	57.61893	56.98365	56.28754	54.79013	55.77805	51.50659		40	1	0.42	1.44
												40	10	0.45	1.22

	0 µM		2 µM				20 µM				Procyanidin B2	Dose Irinotecan	Dose Procyanidin B2	Effect	CI
Irinotecan 0 µM	103.1659	96.07416	93.36292	90.33745	94.29169	92.83958	87.80668	90.34639	87.85094	87.10259		2	2	0.15	0.64
Irinotecan 2 µM	91.49785	86.37304	85.84248	81.87934	85.92677	84.66551	79.95562	76.98348	79.70411	73.74594		2	20	0.22	0.35
Irinotecan 40 µM	52.53591	46.75377	48.48449	44.99082	48.08683	49.35085	44.95138	45.34877	42.95322	41.02083		40	2	0.52	0.89
												40	20	0.56	0.71

	0 µM		1 µM				10 µM				Resveratrol	Dose Irinotecan	Dose Resveratrol	Effect	CI
Irinotecan 0 µM	101.3824	98.6176	94.28089	94.46091	100.27	95.10972	77.72415	75.55059	72.6904	76.42544		2	1	0.08	1.08
Irinotecan 2 µM	84.62663	91.05762	90.33554	91.92896	95.50869	91.69376	77.44589	73.73306	75.562	71.70278		2	10	0.25	1.07
Irinotecan 40 µM	50.49524	50.35021	53.9407	52.60418	55.88917	51.76789	46.67876	44.80098	46.15483	44.5515		40	1	0.46	0.99
												40	10	0.54	0.91

	0 µM		2 µM				20 µM				Urolithin A	Dose Irinotecan	Dose Urolithin A	Effect	CI
Irinotecan 0 µM	98.81404	100.8009	100.0194	95.97951	99.37239	96.90602	78.8684	78.82003	77.17248	77.21111		2	2	0.09	1.17
Irinotecan 2 µM	87.66154	94.2708	87.47607	92.01616	92.20714	90.81812	77.3383	76.23894	70.34416	67.14843		2	20	0.27	0.94
Irinotecan 40 µM	51.7659	57.44156	57.62918		51.51518	59.59987	49.10688	44.68004	44.14899	41.45963		40	2	0.58	0.50
												40	20	0.55	0.84

	0 µM		0.5 µM				5 µM				Fulvestrant	Dose Irinotecan	Dose Fulvestrant	Effect	CI
Irinotecan 0 µM	98.64942	101.3506	95.95047	90.53973	97.79528	94.22552	72.8699	72.60959	73.52505	67.68147		2	0.5	0.10	2.17
Irinotecan 2 µM	86.68812	86.21781	84.51141	94.17123	92.00835	88.69828	66.19802	69.09533	65.16726	63.30956		2	5	0.34	0.86
Irinotecan 40 µM	52.28735	49.84441	48.68421	49.31457	53.23803	50.78833	35.6758	39.0962	34.90703	34.68938		40	0.5	0.49	1.04
												40	5	0.64	0.54

	0 mM		0.4 mM				4 mM				Metformin	Dose Irinotecan	Dose Metformin	Effect	CI
Irinotecan 0 µM	99.59841	100.4016	98.25635	100.0031	97.0297	101.857	87.0002	84.56211	82.62481	78.65089		2	0.4	0.11	1.26
Irinotecan 2 µM	85.86317	92.96757	83.86021	91.73082	90.97932	89.87624	72.19035	74.21095	73.58254	73.92422		2	4	0.26	0.46
Irinotecan 40 µM	54.48353	49.29811	50.65892	54.67714	51.52627	50.73465	43.96557	40.69005	43.10172	41.20882		40	0.4	0.48	1.00
												40	4	0.58	0.57

Table S3. Overall analysis of combinations and combination index values in this study.

	Synergy with chemotherapeutics								
Chemotherapeutic	5-FU	5-FU	Iritonecan	Iritonecan	Iritonecan	Iritonecan	Gemcitabine	Gefitinib	Iritonecan
	WİDR	KM12	SW837	ASPC1	BXPC3	MIAPACA2	MIAPACA2	MIAPACA2	SU8686
Apigenin	yes	no	no	ns	ns	ns	ns	ns	ns
Curcumin	no	no	no	yes	no	no	ns	ns	no
EGCG	yes	no	no	ns	ns	ns	ns	ns	ns
Fisetin	slightly	no	no	ns	ns	ns	no	no	ns
Forskolin	no	yes	no	ns	ns	ns	no	no	ns
Procyanidin B2	no	no	yes	ns	ns	ns	no	no	ns
Resveratrol	no	no	no	no	slight	slight	yes	no	slight
Urolithin A	no	slightly	yes	ns	ns	ns	ns	ns	ns
Fulvestrant	slightly	yes	slight	ns	ns	ns	no	no	ns
Metformin(mM)	slightly	slightly	yes	ns	ns	ns	no	no	ns

*ns: not screened

*no: CI>1

*yes: CI<0.7

*slightly: 1<CI<0.7
